# A Novel Approach to Modeling and Forecasting Cancer Incidence and Mortality Rates through Web Queries and Automated Forecasting Algorithms: Evidence from Romania

**DOI:** 10.3390/biology11060857

**Published:** 2022-06-03

**Authors:** Cristiana Tudor

**Affiliations:** International Business and Economics Department, The Bucharest University of Economic Studies, 010374 Bucharest, Romania; cristiana.tudor@net.ase.ro

**Keywords:** cancer, incidence, mortality, modeling, forecasting, Google Trends, Romania, ARIMA, TBATS, NNAR

## Abstract

**Simple Summary:**

Cancer remains a global burden, currently causing nearly one in six deaths worldwide. Accurate projections of cancer incidence and mortality are needed for effective and efficient policymaking, accurate resource allocation, and to assess the impact of newly introduced policies and measures. However, the COVID-19 pandemic disrupted public health systems and caused a significant number of cancers to remain undiagnosed, thus affecting the quality of official statistics and their usefulness for health studies. This paper addresses this issue by proposing novel cancer incidence/cancer mortality models based on population web-search habits and historical links with official health variables. The models are empirically estimated using data from one of the most vulnerable European Union (EU) members, Romania, a country that consistently reports lower survival rates than the EU average, and are further used to forecast cancer incidence and mortality rates in the country. Research findings have important policy implications, and the novel framework, owing to its generalizability, can be applied to the same task in other countries. Overall, the results indicate a continuation of the increasing trends in cancer incidence and mortality in Romania and thus underline the urgency to change the status quo in the Romanian public-health system.

**Abstract:**

Cancer remains a leading cause of worldwide mortality and is a growing, multifaceted global burden. As a result, cancer prevention and cancer mortality reduction are counted among the most pressing public health issues of the twenty-first century. In turn, accurate projections of cancer incidence and mortality rates are paramount for robust policymaking, aimed at creating efficient and inclusive public health systems and also for establishing a baseline to assess the impact of newly introduced public health measures. Within the European Union (EU), Romania consistently reports higher mortality from all types of cancer than the EU average, caused by an inefficient and underfinanced public health system and lower economic development that in turn have created the phenomenon of “oncotourism”. This paper aims to develop novel cancer incidence/cancer mortality models based on historical links between incidence and mortality occurrence as reflected in official statistics and population web-search habits. Subsequently, it employs estimates of the web query index to produce forecasts of cancer incidence and mortality rates in Romania. Various statistical and machine-learning models—the autoregressive integrated moving average model (ARIMA), the Exponential Smoothing State Space Model with Box-Cox Transformation, ARMA Errors, Trend, and Seasonal Components (TBATS), and a feed-forward neural network nonlinear autoregression model, or NNAR—are estimated through automated algorithms to assess in-sample fit and out-of-sample forecasting accuracy for web-query volume data. Forecasts are produced with the overperforming model in the out-of-sample context (i.e., NNAR) and fed into the novel incidence/mortality models. Results indicate a continuation of the increasing trends in cancer incidence and mortality in Romania by 2026, with projected levels for the age-standardized total cancer incidence of 313.8 and the age-standardized mortality rate of 233.8 representing an increase of 2%, and, respectively, 3% relative to the 2019 levels. Research findings thus indicate that, under the no-change hypothesis, cancer will remain a significant burden in Romania and highlight the need and urgency to improve the status quo in the Romanian public health system.

## 1. Introduction

Cancer remains a primary cause of death worldwide [1] and acknowledged as a growing global burden [2]. Moreover, many healthcare systems in less developed countries are ill-equipped to adequately deal with this burden, and a huge percentage of cancer patients worldwide lack access to timely, high-quality diagnosis and treatment [3]. As of 2020, cancer accounted for approximately 10 million deaths worldwide, or nearly one in six deaths. Furthermore, cancer maintains its place as the second leading cause of death in many nations, trailing only cardiovascular disease [3,4,5,6]. Additionally, the number of cancer diagnoses and fatalities is expected to significantly increase over the next decade, with projections for 2030 indicating 26 million new cancer cases and 17 million cancer deaths per year [7].

Concurrently, cancer is one of the most critical economic and financial burdens that the globe faces today [8]. In the United States alone, national costs of cancer totaled USD 183 billion as of 2015, with projections that include only population growth indicating an increase of 34% by 2030, reaching USD 246 billion [9].

Consequently, with this escalating global burden, cancer prevention and cancer mortality reduction are counted among the most serious public health concerns of the twenty-first century [10]. In particular, the term “primary prevention” refers to measures to reduce the incidence of the disease, whereas “secondary prevention” refers to efforts to diagnose cancer early or to reduce second cancers among cancer survivors [11]. Accurate cancer projections for future time points are paramount for both primary and secondary prevention and are additionally critical for planning future services and resource allocation, as well as establishing and evaluating cancer control programs [12]. However, time series forecasting is a challenging task [13], whereas producing accurate estimates for the future rates of cancer incidence and mortality is additionally complicated due to the short time series available. For example, at the time of the study the Eurostat (i.e., the statistical office of the European Union) database provides statistics for cancer deaths at a European level spanning the period 2011–2018, whereas the World Development Indicators (WDI) database of the World Bank offers data on the mortality rate from cardiovascular disease, cancer, diabetes, or chronic respiratory disease, and thus does not individualize cancer. Additionally, with short series, out-of-sample forecasting accuracy is hard to assess, and time series cross-validation can be difficult to implement [14].

To solve such research obstacles, monitoring health-seeking behavior in the form of public interest indicated by online search queries has emerged as an essential technique for early identification of health problem occurrences throughout certain periods and geographies [15]. This in turn is based on the fact that the internet has grown in importance as a source of health information accessed by the world population [16,17]. As a direct result, Google Trends has become increasingly popular in health and medical research over the past decade [15,18].

**Figure 1:**
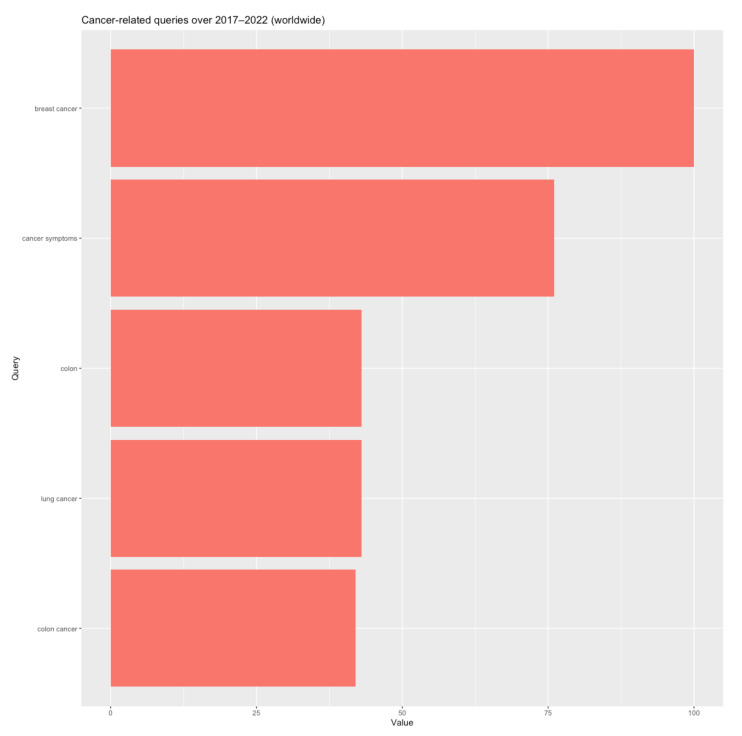
Most common queries related to the search term “cancer”: worldwide (April 2017–March 2022). Source of data: Google Trends. Estimation results using the “gtrendsR” package [19] in R software.

Our data confirm the relevance of web searches for highlighting real occurrences of health problems. Thus, [1] indicates that the three most common types of cancer in 2020, in terms of new cases, were breast cancer with 2.26 million cases, lung cancer with 2.21 million cases, and colon and rectum cancer with 1.93 million cases. Concurrently, as reflected in Figure 1, these were the exact web queries related to the term “cancer” over recent years at the world level, confirming that Internet searches are an accurate reflection of health issue incidences.

**Figure 2:**
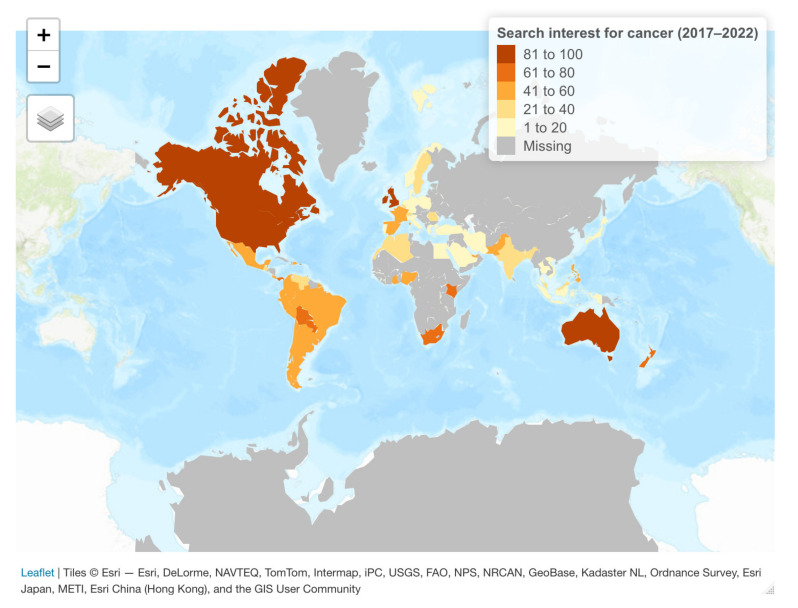
Internet search interest for “cancer” at the world level: (April 2017–March 2022). Source of data: Google Trends. Map is based on estimation results and uses the packages “gtrendsR” [19] and “tmap” [21] in R software.

Moreover, a visualization of the global web-search interest reveals that most normalized searches emerged in countries that also reported the highest age-standardized cancer rates. Of note, to accurately comprehend the geography of search interest for a given keyword, the term should be searched across all the world’s languages. However, Google Trends provides a specific tool capable of dealing with this issue, i.e., “Topics,” which collects all related words, variant spellings, and names in other languages under a single label to help with comprehending topics in a multilingual setting. Topics can thus be particularly effective in combining translations into multiple languages under a single subject [20]. As such, we specified the topic “cancer” when sourcing Google Trends data. Thus, Figure 2 reflects the normalized number of internet searches over the most recent five years, confirming that the highest population interest in the topic “cancer” was encountered in countries including Australia, the US, and Ireland. On the other hand, WHO data confirm that Australia registered the world’s highest age-standardized cancer rate at 452.4 cases per 100,000 people in 2020, followed by New Zealand (422.9), Ireland (372.8), and the United States (362.2).

Additionally, studies increasingly confirm that many cancers remained undiagnosed as a result of healthcare system disruptions caused by the COVID-19 outbreak [22,23,24]. In this context, with official statistics failing to accurately capture the variation in incidence, people’s search interest for specific symptomatology emerges as the most relevant indication of the health problem’s occurrence.

In light of the above considerations, this study sourced Google Trends data to extract information on internet searches for the word “cancer” and employed it as a proxy to forecast cancer incidence rates. Google Trends (www.trends.google.com, accessed on 30 March 2022) is a web-based tool that shows the popularity of a search phrase in a certain location over time. It provides a time series index of the number of Google queries submitted in a given location. The query weight or share is calculated by dividing the overall query volume for a specific search term within a geographic region by the total number of searches in that region throughout the period in question. Following that, the result is scaled from 0 to 100. As a result, the maximum query share of a search phrase for a given period is normalized to 100, reflecting the point when the search was at its most popular. In conclusion, on a scale of 0–100, Google Trends calculates relative search interest (RSI), with 100 reflecting peak interest [25]. Ref. [26] explore the utility of Google Trends data to examine population web searches for cancer screening and conclude that web queries can capture awareness and interest in cancer screening. Thus, Google Trends data may complement traditional data collection and analysis about cancer screening and related interests, providing important scientific possibilities. However, given the aforementioned disruption of public health systems caused by COVID-19 that altered official statistics, we argue that Google Trends data can now be used as a substitute for traditional statistics, which further expands its scientific value.

**Figure 3:**
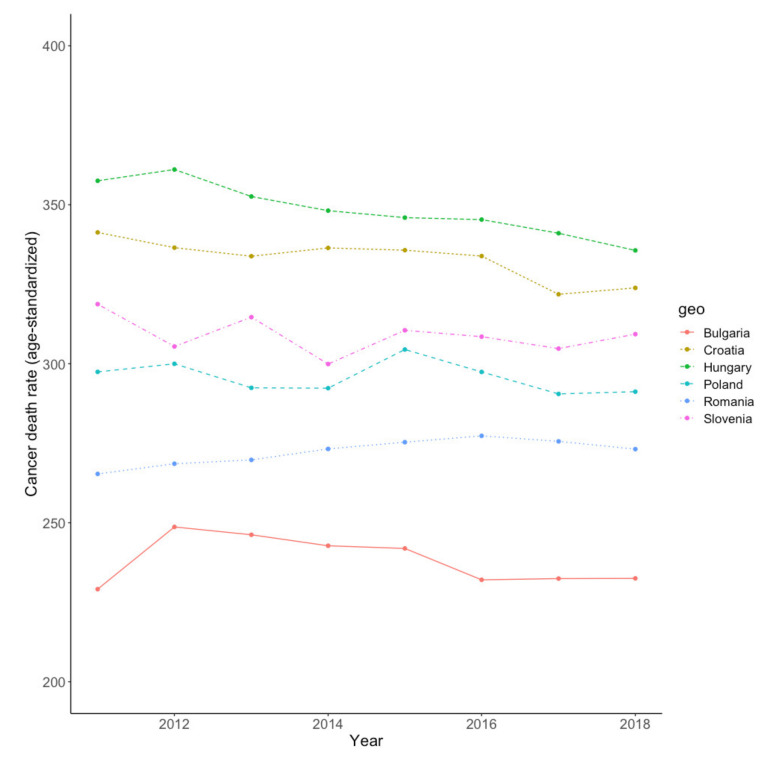
Trends in cancer mortality rates in selected CEE countries (2011–2018). Estimation results. Plot created in R software (“ggplots” function). Source of data: Eurostat.

Of note, among European countries, cancer survival is significantly lower in newer and less developed EU members from Central and Eastern Europe [27]. Higher death rates at the CEE level are caused by two main factors: delayed diagnosis and suboptimal treatment [28], which are in turn related to inefficient and underfinanced public health systems and lower overall economic development [29].The situation is particularly challenging in Romania, which continues to register a divergent trend in mortality rates relative to its EU counterparts, including in the CEE area [30]. Figure 3 shows that the age-standardized mortality rate from all cancers follows an increasing trend in Romania from 2011 to 2018, reflecting the inefficiency of the public healthcare system in the country, whereas most other CEE countries have managed to reverse the trend.

Moreover, the excess mortality from the main types of cancer registered in Romania relative to the EU average is reflected by the difference in the five-year survival rates presented in Table 1. For example, whereas recent statistics show that the survival rate of breast cancer patients has rapidly increased over recent years, due to the availability of early diagnostic tools and treatment [31], Romania still reports significant health gaps, which is heavily influenced by the fact that there is no organized population screening for breast cancer in the country [30].

**Figure 4:**
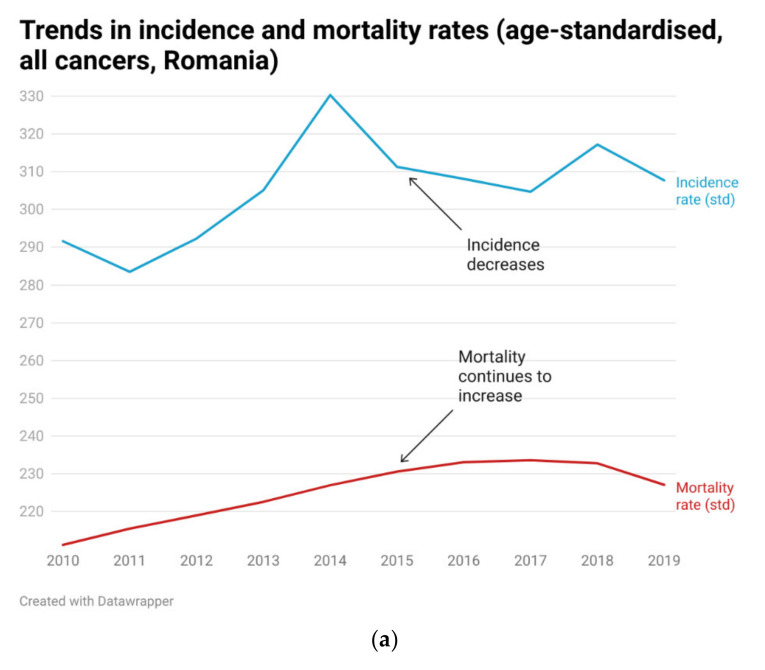

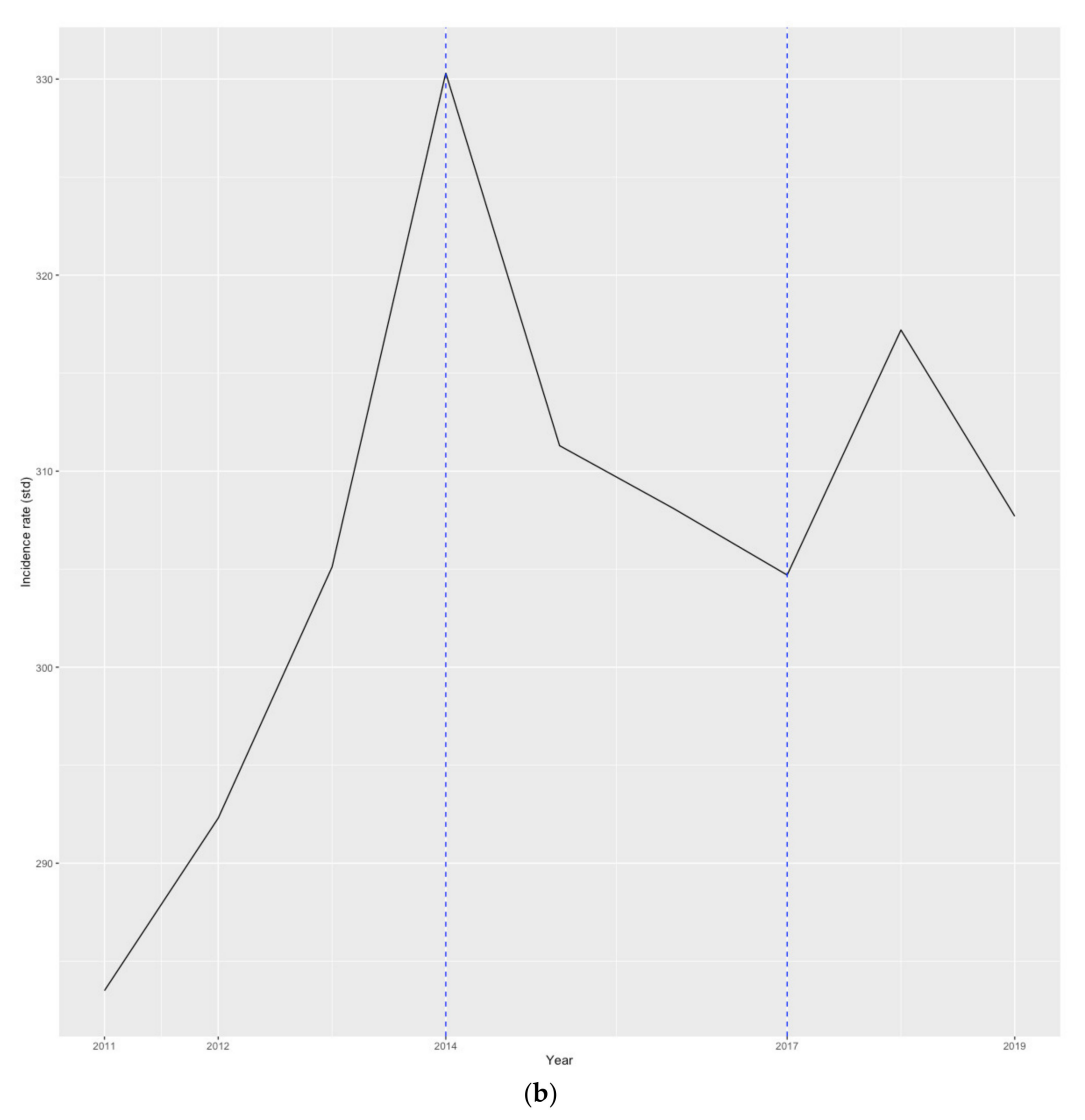
Trends in age-standardized cancer incidence and mortality rates in Romania (2010–2019) (panel **a**); join-points in cancer incidence rate (panel **b**). Source of data: Romanian Ministry of Health (2021) [32]. Chart in panel (**a**) is produced in Datawrapper. Chart in panel (**b**) is produced with the “ggplot” function in R software; join-point regression analysis is performed with the “segmented” package within R software.

A worrisome disaggregation also occurs between cancer incidence and mortality rates registered in Romania, which has not managed to reduce cancer mortality despite periods of decreased incidence and which further highlights the necessity and urgency of better policies aimed at providing an efficient and inclusive public health system (Figure 4, Panel a). Moreover, the implementation of join-point regression analysis, also known as change-point regression or segmented regression analysis [33] to detect changing trends in cancer incidence (see for example [34,35,36,37,38,39]) further confirms that the incidence rate in Romania presents two join points in 2014 and 2017, leading to three periods with a different trend over the analysis period, as follows: a positive trend with a slope coefficient of 14.68 until 2014, a negative trend with a slope coefficient of −8.83 during 2014–2017, and a slightly increasing trend with a slope of 2.05 after 2017 (Figure 4, Panel b). Hence, whereas the incidence follows an increasing trend until the first join-point (i.e., 2014), a reversal is detected thereafter, and a decreasing trend is confirmed over the second segment (i.e., 2014–2017). However, the decreasing trend is reversed thereafter, as the incidence rate presents a subsequent rise. On the other hand, the join-point regression analysis found no join-point in the mortality rate, confirming the disaggregation between the two series.

Patient migration from CEE countries has grown with the implementation of a European Union Directive issued in 2011. According to this directive, European nationals are eligible to use European healthcare services in any of the European member states, and their treatments are covered (at least partially) by their home country’s health insurance system [40]. As a result, a phenomenon called “oncotourism” or “cancer tourism” has emerged, whereas diagnosed patients move away from inefficient Eastern and Central European public healthcare systems, particularly from Romania, toward the private system or the healthcare systems of more developed EU countries [41].

Consequently, accurate predictions for cancer incidences are paramount for early detection and for issuing effective and more inclusive public health policies, especially in the most vulnerable EU members in the CEE area. Additionally, cancer incidence projections are also useful for planning health services and establishing a baseline for evaluating the impact of public health measures [42]. Thus, the main goal of this study is to develop cancer incidence/cancer mortality models, and subsequently to make use of web-search data extracted from Google Trends and its point estimates issued through an array of statistical and machine-learning models to ultimately produce accurate forecasts of cancer incidence and mortality rates, while taking a special focus on a vulnerable CEE country significantly plagued by this disease, i.e., Romania. From a methodological perspective, the robustness of results is assured through various approaches, such as: (i) the estimation of alternative predictive models (statistical and machine-learning); (ii) the assessment of the relative out-of-sample forecasting accuracy through the hold-out forecasting technique; (iii) the estimation of the Diebold-Mariano test for superior forecasting accuracy, and (iv) resampling Google Trends data and employing the sampling average for the web-query index.

Of note, the vast majority of previous research either employs one forecasting method or assesses the predictive ability of concurrent methods by estimating forecasting accuracy metrics. This study implements alternative predictive models, both statistical and machine learning, through automated forecasting algorithms. Moreover, the random sampling issue that arises from using Google Trends data is mitigated through resampling and averaging. Additionally, the forecasting results are defended against the Diebold-Mariano (DM) predictive accuracy test. Hence, various robustness checks confirm the reliability of current findings. Furthermore, the strand of literature on cancer research, particularly with a focus on Central and Eastern Europe, remains thin. Hence, whereas most related studies focus on developed countries, the current research contributes to filling the literature void and is thus concerned with a rather under-investigated EU member, Romania, a country that constitutes an interesting playing field for cancer research due to divergent trends relative to its EU counterparts and plagued by the worrisome phenomenon of “oncotourism”. The proposed method is novel and carries the generalizability advantage, being suitable to further investigate other countries for which official statistics have been heavily affected by the coronavirus pandemic.

Thus, compared to previous studies, the contributions of the current research are threefold: (i) we develop two novel models to explain cancer incidence and cancer mortality rates that embed both official statistics and data on population health-seeking behavior as reflected in internet search habits, whereas most previous studies employ some version of the age-period-cohort model (APC) for the same task; (ii) we propose a robust and integrated approach for web query volume forecasting that includes statistical and machine-learning forecasting methods and assures the robustness of results through multiple model calibration on training and test datasets and estimation of multiple accuracy metrics; and (iii) we apply this novel framework to data from one of the most vulnerable EU members, Romania, a country increasingly defined by the phenomenon of “oncotourism”, whereby diagnosed patients avoid the inefficient national public health system. We additionally provide evidence on the link between internet-seeking behavior and the incidence and mortality of the disease in Romania, thus contributing to the extent of infodemiological literature. Research findings have important policy implications, and the framework, owing to its generalizability, can be applied to the same task in other countries. The novel approach is particularly relevant in the aftermath of the COVID-19 pandemic, which has disrupted public health systems and caused a significant number of cancers to remain undiagnosed, thus affecting the quality of official statistics and their usefulness for health studies.

Results overall indicate a continuation of the increasing trends in cancer incidence and mortality in Romania, with a standardized cancer incidence rate of 313.8 by 2026 and a standardized cancer mortality rate of 233.8 by the same horizon, and thus underline the urgency to change the status quo in the Romanian public health system.

The paper continues as follows. Section 2 presents the data used in model development and explains the integrated method. Section 3 describes the empirical findings that emerge from implementing the novel-forecasting framework on the Romanian data. Section 4 discusses the main findings and, finally, Section 5 concludes the study.

## 2. Materials and Methods

In this study, we sourced annual data on Romanian cancer incidence and mortality rates spanning 2010–2019 from the Romanian Ministry of Health. Next, to develop a model capable of explaining and forecasting these relevant health indicators in the absence of reliable official statistics (which is a worldwide issue caused by the significant number of undetected cancer cases after the onset of the COVID-19 pandemic), we relied on previous infodemiological studies that acknowledge the population’s internet search habits as a reliable proxy for the incidence of a health problem.

The Google Trends platform is a handy tool for determining the popularity of a specific search keyword among a particular demographic. In this study, we extracted the monthly volume of Google queries issued from Romania for “cancer” for the period spanning January 2005–March 2022. It should be acknowledged that Google Trends implements random sampling and uses only a fraction of the entire search data to construct a search index [43] Thus, to overcome the sample instability issue, multiple samples (i.e., 12) were sourced and the average of samples was used to construct the web-query index, instead of only one sample (see [44] for relevant details on the sample bias and its correction). However, it has also been recognized that the Google Trends sampling procedure produces reasonably precise estimates, and consequently, there is often no need to employ more than a single sample [45]. The web query time series contained 207 monthly observations.

The relationship between web searches and the health indicators of interest for Romania was assessed through the linear model given by Equation (1).

*ŷ* = b*X* + a (1)

where *ŷ* is alternatively the cancer incidence rate, and subsequently the cancer mortality rate, and the independent variable is the web-query index.

Additionally, we used both linear and nonlinear statistical and machine-learning techniques, which allowed us to capture most of the properties of the web-query time series and further contributed to avoiding unreliable forecasts. Predictive models can be delineated into two main categories [46,47,48]: statistical and machine learning methods (self-learning systems that can learn from data and continuously increase performance), respectively. Thus, in this study, the autoregressive integrated moving average (ARIMA) model (Equation (2)), the Exponential Smoothing State Space Model with Box-Cox Transformation, ARMA Errors, Trend, and Seasonal Components (TBATS) given by Equation (3), and the neural network autoregression (NNAR) model reflected in Equation (4) were alternatively fitted.

An ARIMA(*p*,*d*,*q*)(*P*,*D*,*Q*)s model, first developed by [49], is given by:

(2) $$(1-\varphi_{1}B-\ldots -\varphi_{p}B^{p})(1-\Phi_{1}B^{s}-\ldots -\Phi_{P}B^{sP}){\left(1-B\right)}^{d}{\left(1-B^{s}\right)}^{D}Y_{t}=(1-\theta_{1}B-\ldots -\theta_{q}B^{q})(1-\Theta_{1}B^{s}-\ldots -\Theta_{P}B^{sQ})\varepsilon_{t}$$

where *s* is the seasonal period, the lowercase and the capital letters represent nonseasonal and seasonal parameters, and $\varepsilon_{t}$ is a random variable with mean zero and the standard deviation σ.

A TBATS model [50] can accommodate complex seasonal behaviors of data [51] and is written as:

TBATS(ω, *p*, *q*, *φ*, {m_1_, *k*_1_}, {m_2_, *k*_2_}, …, {m_T_, *k*_T_}), (3)

where ω is the Box-Cox transformation, *k* is the number of harmonics used for the seasonal trait, and *φ* is the dampening parameter.

Artificial neural networks (ANNs) are capable of simulating complicated real-world systems while properly accounting for nonlinearities [52]. Lagged values of time series are frequently utilized as inputs in an ANN structure when fitting time series data, which is then known as neural network autoregression (NNAR) [53] (Munim et al., 2019). As in [29,54], the NNAR model is written as:

(4) Y=f(H)=f(W*X+B),X=[y(t−1),y(t−2),…,y(t−p)]

where *Y* stands for the output vector, *f* is the activation function, *H* is the vector of n nodes in the hidden layer, *W* is the weight matrix between the input and hidden layers, *X* is the vector of inputs (i.e., the lagged values of the actual observations), and *B* is a bias vector.

All estimations are automated and performed in R software via dedicated algorithms included in the “forecast” package [55]. To implement the method robustly, the series of length N = 207 was first split into a training set (containing 187 observations) for in-sample fit purposes and a testing set (containing the last 20 observations) on which the models that reported the best fit on the training set were further estimated and their out-of-sample forecasting ability assessed. Lastly, we assessed the forecast accuracy of alternative predictive models by estimating both scale and scale-free accuracy metrics.

First, let us define the forecast error of a candidate model as:

(5) $$e_{T+h}=y_{T+h}-{\hat{y}}_{T+h|T}$$

where {*y_1_*,…,*y_T_*} is the training set data and {*y_T_*_+*1*,_*y_T+2_*,…} is the test-set data.

Then, the following forecasting accuracy metrics are computed as:

Mean absolute error:

(6) $$MAE=\sqrt{\frac{1}{N}\sum_{i=1}^{N}\left|y_{i}-{\hat{y}}_{l}\right|}$$

Root mean squared error:

(7) $$RMSE=\sqrt{\frac{1}{N}\sum_{i=1}^{N}{\left(y_{i}-{\hat{y}}_{l}\right)}^{2}}$$

Mean absolute percentage error:

(8) $$MAPE=mean(|p_{t}|)$$

where: $p_{t}=\frac{100e_{t}}{y_{t}}$ Mean absolute percentage error:

(9) $$\mathrm{MASE}=\mathrm{mean}(|q_{j}|)$$

where *qj* is given by as: $q_{j}=\frac{e_{t}}{\frac{1}{N-1}\sum_{i=2}^{N}|y_{t}-y_{t-1}|}$*f* when the series is non-seasonal and by: $q_{j}=\frac{e_{t}}{\frac{1}{N-m}\sum_{i=m+1}^{N}|y_{t}-y_{t-m}|}$ when the time series is seasonal.

**Figure 5:**
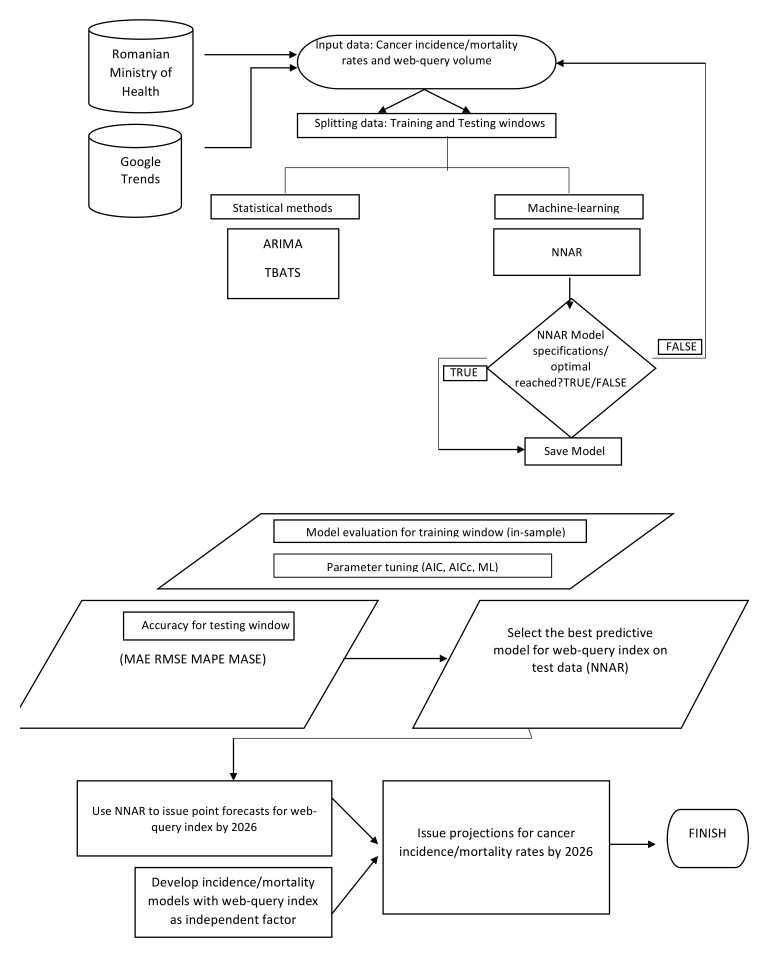
The integrated framework for modeling and forecasting cancer incidence and mortality rates.

Lastly, all predictive models were fitted to the entire series of length N and point forecasts for the web query index for the following 4 years (i.e., a 48-month forecasting horizon) were produced by the overperforming method in the out-of-sample setting. Forecasted values were then fitted into the incidence/mortality models developed by estimating Equation (1), which then issued the expected values for standardized cancer incidence and mortality rates corresponding to the forecasting horizon. Figure 5 reflects the integrated method employed in this study and implemented in R software.

## 3. Results

### 3.1. Relationship between Related Web Queries and the Age-Standardized Cancer Incidence/Cancer Mortality Rate in Romania

**Figure 6:**
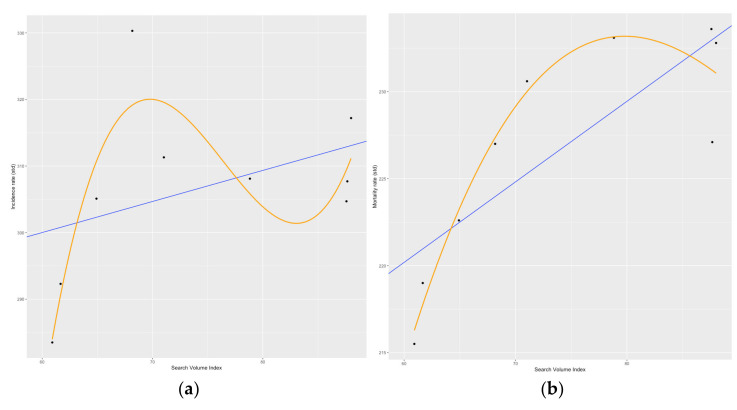
The relationship (linear—blue line, polynomial—orange line) between related web queries and the age-standardized cancer incidence rate in Romania (panel **a**). The relationship (linear—blue line, polynomial—orange line) between related web queries and the age-standardized cancer mortality rate in Romania (panel **b**). Source of data: Romanian Ministry of Health [32]. All estimations were performed in R software; plots were created in R software (i.e.,“ggplot” function).

The best-fit linear model between the vector of web-query volume and the cancer incidence/cancer mortality rate is reflected in Figure 6, panels (a) and (b), respectively. Both representations highlight a positive link between the web-search interest and the variables reflecting the incidence and mortality of the disease. Additionally, both equations show a similar slope coefficient, equal to 0.47 in the incidence rate model, and equal to 0.46 in the mortality rate model.

### 3.2. Results from Modeling and Forecasting the Web-Query Index

Table 2 reports the estimated accuracy measures for the test-set data containing topic searches for “cancer” submitted in Romania that were issued through the statistical and machine-learning predictive models. Results indicate that NNAR has been able to accurately capture variations in data and thus provide the best forecast for the web query index over the testing window.

To assess the forecasting superiority of the feed-forward neural network autoregression model, we estimated the Diebold-Mariano (DM) test [56,57] to examine any significant differences between forecasts produced by NNAR and the second best-performing model (ARIMA). The DM test result (estimated with the “dm.test” function within the “forecast” package in R software) confirmed that there was a significant difference between the distribution of errors from ARIMA and NNAR, thus ensuring the forecasting superiority of the machine-learning method.

**Figure 7:**
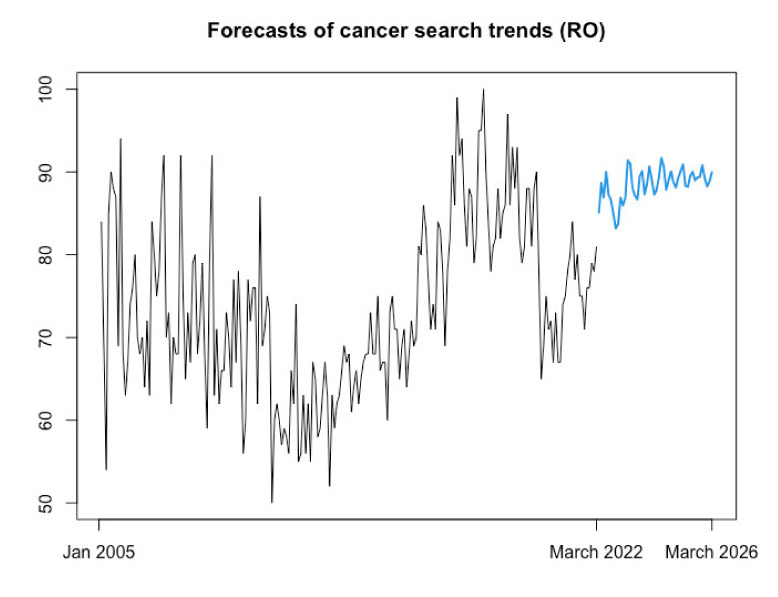
Forecasted trend over April 2022–March 2026 (48 months) for web queries for the term “cancer” in Romania issued with NNAR (12,6). Source: estimation results. Model information: average of 20 networks, each of which is a 12-6-1 network with 85 weight options.

We next employed the best-performing model in terms of out-of-sample forecasting accuracy (i.e., NNAR) to produce the expected web-query volume in Romania for the next 48 months (4 years), corresponding to the period spanning April 2022 to March 2026. Figure 7 reflects the estimation results, showing (in blue color) the point estimates produced by NNAR for April 2022–March 2026. Of note, estimations corresponding to the 48-month forecasting horizon indicated a continuation of the increasing trend in web searches related to “cancer” issued from Romania.

### 3.3. Forecasts of Cancer Incidence and Cancer Mortality Rates in Romania over 2022–2026

Lastly, we put together the linear relationship model estimated before and the point estimates produced by the neural network autoregression model (NNAR) to estimate the cancer incidence and cancer mortality rate for the next four years. It is important to underline that all estimations are based on the status quo hypothesis, implying that these projections are expected if no changes in public health policy are implemented. Projected values for the two health indicators are centralized in Table 3.

We notice that estimations issued through the incidence and mortality models and based on NNAR projections of the related web-search index reflect a continuation of the increasing trends in cancer incidence and mortality in Romania, underlining the urgency to change the status quo in the Romanian public health system. Estimates thus indicate a standardized cancer incidence rate of 313.8 by 2026 and a standardized cancer mortality rate of 233.8 by the same horizon, increasing from levels of 307.7 and 227.1, respectively, registered in 2019.

## 4. Discussion

Approximately 10 million deaths have been attributed to cancer in 2020, or nearly one in six deaths. Concurrently, many cancer patients worldwide still lack access to timely, high-quality diagnosis and treatment. Within the EU, CEE countries consistently report higher mortality rates, mainly as a result of delayed diagnosis and suboptimal treatment. For Romania, statistics are particularly worrisome, with the country reporting significantly lower survival rates for all main types of cancer and also a divergent trend in mortality rates relative to its EU counterparts that have managed to reverse the increasing trend. Consequently, “oncotourism” is especially characteristic of Romania, as diagnosed patients move away from the inefficient public healthcare system toward the private system or the healthcare systems of more developed EU countries.

Consequently, accurate predictions of cancer incidence and mortality rates are keys to informing policymakers and assisting in the policymaking process. The main goal of this study is to develop a robust model capable of capturing the evolution of cancer incidence and mortality rates and forecasting their evolution. Concurrently, we build on [58] and acknowledge that over the past decade, the use of internet data has become an important aspect of health informatics, with online sources becoming more accessible and offering data that can be used to analyze and forecast human behavior. As a result, we also agree with [59] that data from tracking online information seekers’ behavior is useful in public health surveillance and research.

Thus, in model development, the study made use of web-search data extracted from Google Trends, which was introduced as an independent variable in light of previous studies that acknowledge the population’s internet search habits as a reliable proxy for health problem occurrence. Moreover, this approach overcomes the current issue of the unreliability of official statistics, caused on one hand by the numerous undiagnosed cases after the COVID-19 outbreak and, on the other hand, by the unavailability or tardiness of treatment for diagnosed patients, directly affecting the rate of incidence and mortality. Concurrently, point estimates for the web-query index were issued through the best performing predictive model over the test-set (i.e., out-of-sample), after an assessment of the in-sample fit and out-of-sample forecasting accuracy of various statistical and machine-learning models (ARIMA, TBATS, and NNAR) had been performed via several accuracy metrics (MAE, RMSE, MAPE, and MASE). Estimations indicated that NNAR was the most capable of capturing the time series characteristics and of producing the most accurate estimates. Consequently, estimations for the web-query index were automatically produced with the NNAR model and sourced into the incidence/mortality models that have been previously developed. Ultimately, forecasts of cancer incidence and mortality rates in Romania by 2026 were issued, indicating a continuation of the increasing trend for both variables. Our results are in line with projections of [32] the Romanian Ministry of Health (2021), confirming the ascendant trend, although our point estimates fall below the public ministry’s predictions, indicating more conservative increasing rates.

Future predictions depend on multiple assumptions, most importantly on the status quo (i.e., no-change) hypothesis of the Romanian public health system. Similarly, forecasts do not consider the impact of relevant changes in impact factors, such as potential changes in smoking prevalence at the national level, changes in obesity, changes in alcohol consumption, changes in nutrition habits, increased funding of the public health system, increased screening, HPV vaccination, etc. As a result, we agree with [60] that it is critical to review predictions at regular intervals to incorporate the most recent trends in the data. However, similar to [42], we reason that current estimates do provide a useful baseline for the planning of cancer resources and for evaluating the impact of any changes produced in impact factors as a result of newly introduced public health policies and measures. Furthermore, as with most research, this study suffers from other limitations. Mostly, the use of Google Trends data does carry some vulnerabilities that should be acknowledged, including the construction of the search index itself [61]. As a consequence, the long-run stability of the time series is heavily dependent on the data’s time frame and frequency. As per [61], this study used monthly data that was best able to accurately capture the long-term trend. Moreover, random sampling is an inherent bias in Google Trends data [44]. However, this issue is particularly troublesome in forecasting when dealing with topics that are less frequently searched for, which is not the case in the current study. Additionally, the performed resampling further mitigated the bias. Nonetheless, it should be acknowledged that this approach merely minimizes the bias and does not eliminate it. Finally, it should be mentioned that results should be interpreted with care, considering that data reflect the search habits of the population that has Internet access, which in turn depends on income level and other socioeconomic factors. Overall, I argue that the popularity of the search topic, together with the resampling strategy and previous studies that reinforce the usefulness of web search data as a powerful predictive instrument [44] does allow for confidence in the current research findings.

In addition, it should also be mentioned that the link between cancer variables and the web-query index has been assessed through the classical regression model. However, it has been increasingly acknowledged that the neutrosophic regression model [62,63,64,65], which issues the parameters in the indeterminacy interval rage, can be more efficient in the uncertainty environment than the classical regression model [66]. Thus, the assessment of the historical link between cancer incidence/cancer mortality rates and the web search index through the neutrosophic regression model constitutes a good avenue for future research. The implementation of the proposed method on data specific to different gender and age groups, as well as to geographic regions of the country, could also reveal particularly vulnerable groups and/or areas and offer relevant information to policymakers.

From a policy perspective, the findings highlight that cancer will continue to be a significant burden for Romania, which should be carefully planned for. Complementarily, results indicate the need for better policies aimed at mitigating main risk factors such as smoking, alcohol consumption, obesity and overweight, unhealthy nutrition, lack of physical exercise, etc., and at increasing the financing and efficiency of the public health system by allocating future resources for cancer research, treatment, and prevention.

## 5. Conclusions

In conclusion, this study developed two novel cancer incidence/cancer mortality models based on population web-search habits and historical links with official health variables. The models were empirically estimated using data from one of the most vulnerable European Union (EU) members, Romania, and further used to forecast cancer incidence and mortality rates in the country by employing estimates for the web-search query index issued through the best performing out-of-sample forecasting method (NNAR). Research findings have important policy implications, and the novel framework, owing to its generalizability, can be applied to the same task in other countries. It provides the important advantage of overcoming a current issue related to the quality of official statistics in the aftermath of the COVID-19 pandemic that disrupted public health systems and caused a significant number of cancers to remain undiagnosed. Overall, the results indicate a continuation of the increasing trends in cancer incidence and mortality in Romania and thus underline the urgency to change the status quo in the Romanian public health system.

## Figures and Tables

**Table 1:** The five-year survival rates from main types of cancer (Romania versus EU26).

Type of Cancer	5-Year Survival Rate	
	Romania	EU26
Lung	11%	15%
Breast	75%	83%
Prostate	77%	87%

Source of data: Romanian Ministry of Health (2021) [32].

**Table 2:** Accuracy measures for the out-of-sample (test-set) forecasting performance.

Predictive Model	MAE	RMSE	MAPE	MASE
ARIMA	4.21	5.16	5.76	0.61
NNAR	3.96	4.71	5.32	0.57
TBATS	4.60	5.73	6.54	0.74

**Table 3:** Forecasted values for cancer incidence and cancer mortality rates.

Year	Incidence Rate (Projected, Standardized)	Mortality Rate (Projected, Standardized)
2023	308.7	228.8
2024	313.0	233.0
2025	313.6	233.6
2026	313.8	233.8

## Data Availability

Data employed in this study are publicly available from the Eurostat database, from Google Trends, and the Romanian Ministry of Health.
